# Searching for the optimal number of response alternatives for the distress scale of the four-dimensional symptom questionnaire

**DOI:** 10.1186/s12888-019-2070-2

**Published:** 2019-03-29

**Authors:** Jan van Bebber, Johanna T. W. Wigman, Rob R Meijer, Berend Terluin, Sjoerd Sytema, Lex Wunderink

**Affiliations:** 1 University Medical Center Groningen, Interdisciplinary Center Psychopathology and Emotion Regulation (ICPE), University of Groningen, Groningen, The Netherlands; 2 Department of Education and Research, Friesland Mental Health Services, Leeuwarden, The Netherlands; 3 University Medical Center Groningen, Rob Giel Research Center (RGOc), University of Groningen, Groningen, The Netherlands; 4 Department of Psychometrics and Statistics, University of Groningen, Groningen, The Netherlands; 5 Department of General Practice and Elderly Care Medicine, Amsterdam Public Health Research Institute, Amsterdam UMC, Vrije Universiteit Amsterdam, Amsterdam, The Netherlands; 6 Medizinische Klinik mit Schwerpunkt Psychosomatik, Charité - Universitätsmedizin Berlin, Charitéplatz 1, 10117 Berlin, Germany

**Keywords:** Recoding, IRT, GRM, Scale validity, 4DSQ

## Abstract

**Background:**

The Four-Dimensional Symptom Questionnaire (4DSQ) is a self-report questionnaire designed to measure distress, depression, anxiety, and somatization. Prior to computing scale scores from the item scores, the three highest response alternatives (‘Regularly’, ‘Often’, and ‘Very often or constantly present’) are usually collapsed into one category to reduce the influence of extreme responding on item- and scale scores. In this study, we evaluate the usefulness of this transformation for the distress scale based on a variety of criteria.

**Methods:**

Specifically, by using the Graded Response Model, we investigated the effect of this transformation on model fit, local measurement precision, and various indicators of the scale’s validity to get an indication on whether the current practice of recoding should be advocated or not. In particular, the effect on the convergent- (operationalized by the General Health Questionnaire and the Maastricht Questionnaire), divergent- (operationalized by the Neuroticism scale of the NEO-FFI), and predictive validity (operationalized as obtrusion with daily chores and activities, the Biographical Problem list and the Utrecht Burnout Scale) of the distress scale was investigated.

**Results:**

Results indicate that recoding leads to (i) better model fit as indicated by lower mean probabilities of exact test statistics assessing item fit, (ii) small (<.02) losses in the sizes of various validity coefficients, and (iii) a decrease (DIFF (SE’s) = .10–.25) in measurement precision for medium and high levels of distress.

**Conclusions:**

For clinical applications and applications in longitudinal research, the current practice of recoding should be avoided because recoding decreases measurement precision for medium and high levels of distress. It would be interesting to see whether this advice also holds for the three other domains of the 4DSQ.

## Background

The Four-Dimensional Symptom Questionnaire (4DSQ) developed by Terluin [1] is a self-report questionnaire developed in the Netherlands to distinguish symptoms of non-specific general distress from depression, anxiety, and somatization. In the Netherlands, the 4DSQ is widely used in primary (mental) health care settings, and the questionnaire has been translated into English [2], Polish [3], Turkish [4] and other languages (see: www.4dsq.eu). Although initially developed for primary care settings, its validity has also been demonstrated in working populations [5] and ambulant mental health services [6]. Terluin et al. [7] found that the scores on the four scales can be described adequately by unidimensional (common) factor models, and all four scales were found to be invariant with respect to gender, age, and educational level of respondents [8].

Most practitioners working with the 4DSQ found the distress scale most useful and important. This and the fact that the items of this scale were to be used in an adaptive online test battery [9] is also the reason why this article solely focuses on the distress scale of the 4DSQ. The scale comprises sixteen items that express symptoms of nonspecific psychological distress. Respondents have to indicate the frequency of specific symptom experiences during the past week on a five-point scale (‘No’, ‘Sometimes’, ‘Regularly’, ‘Often’, and ‘Very often or constantly’). The reason for using five response categories is that respondents indicated a preference for making finer distinctions than “not present”, “sometimes”, and “constantly present”. However, in practice, the three highest item scores (2–4) are usually recoded to 2. According to the author of this questionnaire, this practice should minimize the influence of extreme responding on scale scores. Until now, the effect of this transformation on the psychometric quality of this scale is unknown. The aim of this paper was to investigate the effect of recoding on the reliability and validity of the 4DSQ using item response theory [10].

### Optimal number of response alternatives: Existing research

Many articles have been devoted to the topic. Cox’ [11] review represents an important contribution. The notion of *signal and noise* was central in this paper. On the one hand, one may strive for maximum refinement of the response scale in order to enable transmission of maximum amount of information in terms of variation. On the other hand, respondents must be capable of using these refinements in a proper way; otherwise, more refinements induce non-systematic variance (i.e., measurement error). To make things even more complicated, this trade-off between signal and noise is probably different for various kinds of items. Additionally, respondents may differ in (i) the way they interpret and use the different alternatives and (ii) in their capacity to distinguish more alternatives in a reliable way. Both aforementioned inter-individual differences increase the noise component in the response data. Although Cox stated that “(…) there is no single number of response alternatives for a scale which is appropriate under all circumstances”, he formulated four recommendations for applied research.

First, scales with only two or three response options are inadequate because these scales are not capable of transmitting much information. Second, using more than nine alternatives does not pay off either. Third, an odd number of alternatives are preferable, assuming that a neutral positon makes sense. Fourth, comprehensible instructions and labeling of response alternatives are crucial.

Three other references that were noted in Cox’s review are also worth mentioning. Cronbach [12] warned that increasing the number of response alternatives in order to achieve a higher reliability of the scale scores may actually facilitate response sets, such as extreme responding, and thus diminishing scale validity. Jacoby and Matell [13] reported that collapsing response alternatives into two or three response categories had a small effect on the reliability and the validity coefficients of a scale. Based on a high positive correlation between respondents’ use of extreme positive and negative responses on the same attitude scale, Peabody [14] concluded that scale scores would partially be reflective of idiosyncratic response sets of individuals.

More recently, Lozano, Garcia-Cueto, and Muniz [15] found in a simulation study that both reliability and construct validity (operationalized as percentage of explained variance by the first principal component) improved with increasing numbers of response alternatives, but that the gains beyond seven options were negligible. In an experimental study, Maydeu-Olivares, Kramp, Garcia-Forero, Gallardo-Pujol, and Coffman [16] found that increasing the number of response alternatives (they used 2, 3, and 5 options) had the following effects: measures of reliability (operationalized as coefficient alpha (CTT) or test information (IRT)) increased, model fit deteriorated, and that convergent validity was not effected by utilizing more response options. In another experimental study, Hilbert et al. [17] found that different response formats (dichotomous, a five-point Likert scale, and a 100 mm Visual Analogue Scale) elicited additional dimensions in response behavior not intended to be measured by questionnaire developers. They concluded that using five-point Likert and 100 mm Visual Analogue Scale as alternatives to dichotomous scoring resulted in additional dimensions to the main dimension found for dichotomous scores. One possible explanation for this phenomenon is extreme response bias. In conclusion, (i) many of the conducted studies focus on a very limited number of psychometric indicators and (ii) studies that shed light on the influence of this factor on various types of validity are rare.

### Aims of this study

Since the current practice of recoding 4DSQ item scores prior to computing scale scores is based on clinical intuition, in this paper, we investigated whether we could find empirical support for this routine. We compared both scoring schemes using the following criteria:

(i) measurement precision across the distress scale;

(ii) the convergent validity of the scale, operationalized as the correlation with the General Health Questionnaire (GHQ) [18, 19] and the Maastricht Vital Exhaustion Questionnaire (MQ) [24];

(iii) the discriminant validity of the scale, operationalized as the correlation of the 4DSQ distress scores with the scores on the Neuroticism scale of the NEO Five Factor Inventory (NEO-FFI) [20];

(iv) the predictive validity of the scale, operationalized as the correlation of the 4DSQ distress scores with the scores on the Biographical Problem List (BPL) [21], feelings of work-related exhaustion, distance and competence based on the Utrecht Burnout Scale (UBOS) [22], and sick leave.

## Methods

### Participants

We used data from three samples in which the 4DSQ was assessed in our analyses. The first sample comprised 1793 clients who visited their General Practitioner (GP) in the Netherlands between 2004 and 2011 for psychological complaints. We decided to delete the records of 776 respondents because they had missing values on some of the distress items. Having no respondents with missing values simplified the IRT analyses and a sample size of more than 1000 respondents is still large enough to warrant stable parameter estimates. Mean age was 40.2 years (SD = 14.9, age range 11–85), and 63.3% were female. We used this sample for calibration, assessing model fit and computing local measurement precision. Hence, in the remainder of this article, we refer to this sample as *calibration sample.*

The second sample comprised 55 GP clients of whom the GP suspected to have a mental health problem. Consultations took place in GP practices in the Netherlands in 1998. The inclusion criteria for this sample are thoroughly described in [1]. Mean age was 40.4 (SD = 10.6, age range 17–86 years), and 52.7% were female. We used this sample for assessing the convergent validity (CV) of the distress scale; hence, in the remainder of this article, we refer to this sample as *CV sample*.

The third sample comprised 429 GP-clients who participated in the Effectiveness of a Minimal Intervention for Stress-related mental disorders with Sick leave (MISS) study [23]. Inclusion criteria were (i) having a paid job, (ii) sick leave for no longer than three months, and (iii) elevated levels of distress. Mean age was 40.3 years (SD = 9.3, age range 20–60). Approximately 67% of respondents were female. There were four different measurements: baseline (t_0_; 2003–2005), and three follow-up measurements (2004–2006). The first follow-up measurement was after two months (t_1_), the second after six months (t_2_) and the third after twelve months (t_3_). At each time point, respondents filled out the 4DSQ and various indicators of social and occupational functioning (for further details see below). We used this sample to access the discriminant and predictive validity of the scale and refer to this sample in the remainder of this article as *MISS sample*.

### Instruments

#### Psychometric properties 4DSQ distress scale

The reliability of the scale, operationalized as coefficient alpha, equaled .90 in primary care settings as well as in mental outpatient settings [2, 5]. Also, research indicated that the scale scores could be adequately described by a unidimensional (common) factor model [7], as was the case for the scores on the three other 4DSQ scales. For the higher order structure, a model with four factors fit the data significantly better than alternative structures, wherein, for example, depression items were allowed to load on two distinct factors [7]. Furthermore, all four scales were found to be invariant regarding age, gender, and educational level of respondents [8].

In addition, the structure of the nomological network of the distress scale was in accordance with theoretical expectations. Regarding convergent content validity, correlations of moderate size were found with other nonspecific measures of distress: r = .58 using the General Health Questionnaire, and r = .46 for the Maastricht Questionnaire.

With respect to predictive validity, negative associations with various measures of occupational (*R*^2^ = .29) and social (*R*^2^ = .31) functioning [7] were reported. Furthermore, scores on the distress scale were found to be predictive for the occurrence of psychosocial problems (R^2^ = .30), and the history of stress-inducing life events (R^2^ = .11) [7] can be (post) predicted by the scores on the distress scale.

##### General health questionnaire (GHQ)

The GHQ [18, 19] consists of 30 nonspecific mental [24] health symptoms, which are rated on a 4-point Likert scale ranging from ‘Not at all’ to ‘Much more than usual’. Similar to the 4DSQ, two types of scoring rules do exist (0–3 or 0–1). Reliability, operationalized as coefficient alpha is approximately .90 in various populations. We decided to use the binary coding in this study, because more than three response options could possibly trigger extreme response bias in respondents (B. Terluin, personal communication, October 12, 2016).

##### Maastricht vital exhaustion questionnaire (MQ)

The MQ [19] consists of 21 dichotomously scored nonspecific symptoms of mental health that reflect cardiac dysfunction. Cronbach’s alpha equaled .89 and significant associations with future angina and myocardial infarction have been found [25].

##### Neuroticism (NEO-FFI)

The Neuroticism scale of the revised and shortened NEO [20] consists of twelve 5-point Likert items. The internal consistency (alpha) of the scale is generally above .80, the precise value depending on the population in which it is deployed. The test-retest reliability of the scale equaled .80, and both, the convergent and divergent (discriminant) validity have been rated as good by the Dutch commission of test affairs [26].

##### Obtrusion of daily chores and activities

Clients who participated in the MISS study [23] were asked whether they had trouble performing daily chores and activities. Response options were ‘No problems’, ‘Some problems’, and ‘Unable to perform’. Because only ten (in the third wave) and seven (in the fourth wave) clients choose the last category, we decided to merge this option with the mid-category ‘Some problems’. For both scoring rules, we computed the proportion of explained variance in this dichotomy, using Nagelkerke’s R-square (an adjusted measure of explained variance for categorical variables in logistic regression).

#### Biographical problem list (BPL)

The BPL [21] comprises eighteen problem statements with response options ‘Yes’ or ‘No’. Instead of using one total score based on all items, we decided only to those statements that do not refer to physical functioning, because physical functioning items seem not directly relevant to psychological distress. Furthermore, in order to create criterion measures with homogenous content, we decided to spilt the remaining items in a subscale consisting of six relational problem statements (alpha = .57) and eight general problem statements (alpha = .65). The chosen statements can be found in Table 6 in the Appendix.

##### Utrecht burnout scale (UBOS)

The UBOS [22] measures three components of burnout: exhaustion, distance and competence. Each component is operationalized by four to six symptoms, and respondents have to rate the frequency of occurrence on a 7-point Likert scale. Internal consistencies of the scales range from .75 to .88, and a factor model with three factors shows acceptable fit (CFI: .93, RMSR: .05). Regarding convergent validity, the exhaustion scale correlates with need for recovery (.75) and sleep problems (.45), the distance scale with role conflict (.45), and the competence scale with loss of motivation (−.37). Significant correlations (−.16–.27) with sick leave are indicative of the predictive validity of the scales.

### Measures, measurements, and types of scale scores

Note that the BPL was assessed two times, six and twelve months after baseline. The UBOS and our registration of sick leave was assessed only once, twelve months after baseline. In the remainder of this article, we refer to the first follow-up measurement (after six months) as short-term, and to the second follow-up measurement (after twelve months) as long-term. Descriptive statistics for all measures in our study on all measurement occasions (baseline, six months and twelve months after baseline) may be found in Table 7.

For both scale scores, that is, the original five-point item response scale (0–4) and the recoded three-point item response scale, we expected positive relationships with all criterion measures (GHQ MQ, NEO-FFI, BPL, UBOS, & sick leave), indicating that higher levels of distress correspond to higher scores on the criterion measures. For all criterion measures except for the NEO-FFI, which we use as an indicator of discriminant validity, higher values of correlation coefficients indicated the more valid scale scores. For the NEO-FFI, the lower correlation is indicative of the more valid scale score.

### Item response theory

In the clinical field, Item Response Theory (IRT) models are increasingly becoming the standard way of evaluating the quality of measurement instruments both for linear and adaptive questionnaires [27–29]. IRT offers several advantages over classical test theory for reliability estimation and investigating construct validity. With respect to reliability, measurement precision can be assessed conditional on the trait value that is being measured (that is, locally) instead of using an index like Cronbach’s alpha that provides an overall estimate of the reliability of the scale. Note that this overall index may be imprecise for some scale intervals. More specifically, it is often too high for extreme values. Furthermore, regarding the construct validity of the scale, the correctness of the proposed ordering of response alternatives can be evaluated [30]. Another advantage of IRT is that IRT scores are more spread out than simple sum scores, especially in the tails of the distribution [10]. This characteristic may prove advantageous when investigating relationships with other important variables in the nomological network.

Despite the aforementioned strengths, the chosen IRT model must fit the item scores reasonably well, and item scores have to fulfill certain assumptions. Most important, item scores have to be uncorrelated (locally independent) once item scores are controlled for differences among respondents on the latent trait [10]. More specifically formulated for our case, the items of the distress scale have to be essentially uncorrelated when item scores are controlled for differences among respondents in levels of distress. Two item pairs of the distress scale violated this assumption of local independence due to common item content. Both items of the first pair refer to sleeping problems and items of the second pair both to residual effects of traumatic experiences [7]. Therefore, we decided to remove the item of each pair with the lowest loading on the first common factor.

In this study we used the graded response model (GRM) [31, 32] to compare both scoring rules in terms of model fit (of individual items and at scale level), and in terms of local measurement precision. The GRM is often used to analyze clinical and personality scales. It is a generalization of the two parameter logistic model [10]. Polytomous items are treated as series of k-1 dichotomies, where *k* represents the number of response options. Each logistic function (so called operating characteristic curve, OCC) models the probability of a response in or above a certain category, conditional on the trait or characteristic that is being measured by the scale (distress in our case). Two types of parameters define each item. The first parameter is the slope parameter. This parameter expresses how quickly a response above a certain category becomes more likely with increasing levels of distress. The second parameters are *k*-1 category threshold parameters. These parameters denote the points on the distress continuum where the probability of responding above a certain category becomes more likely than choosing the given category. From the *k*-1 OCCs, k category response curves (CRCs) can be deduced. The CRCs display the probability of choosing a certain response option, given a certain distress level. For each item, these sum up to one at any point on the latent continuum.

**Fig. 1:**
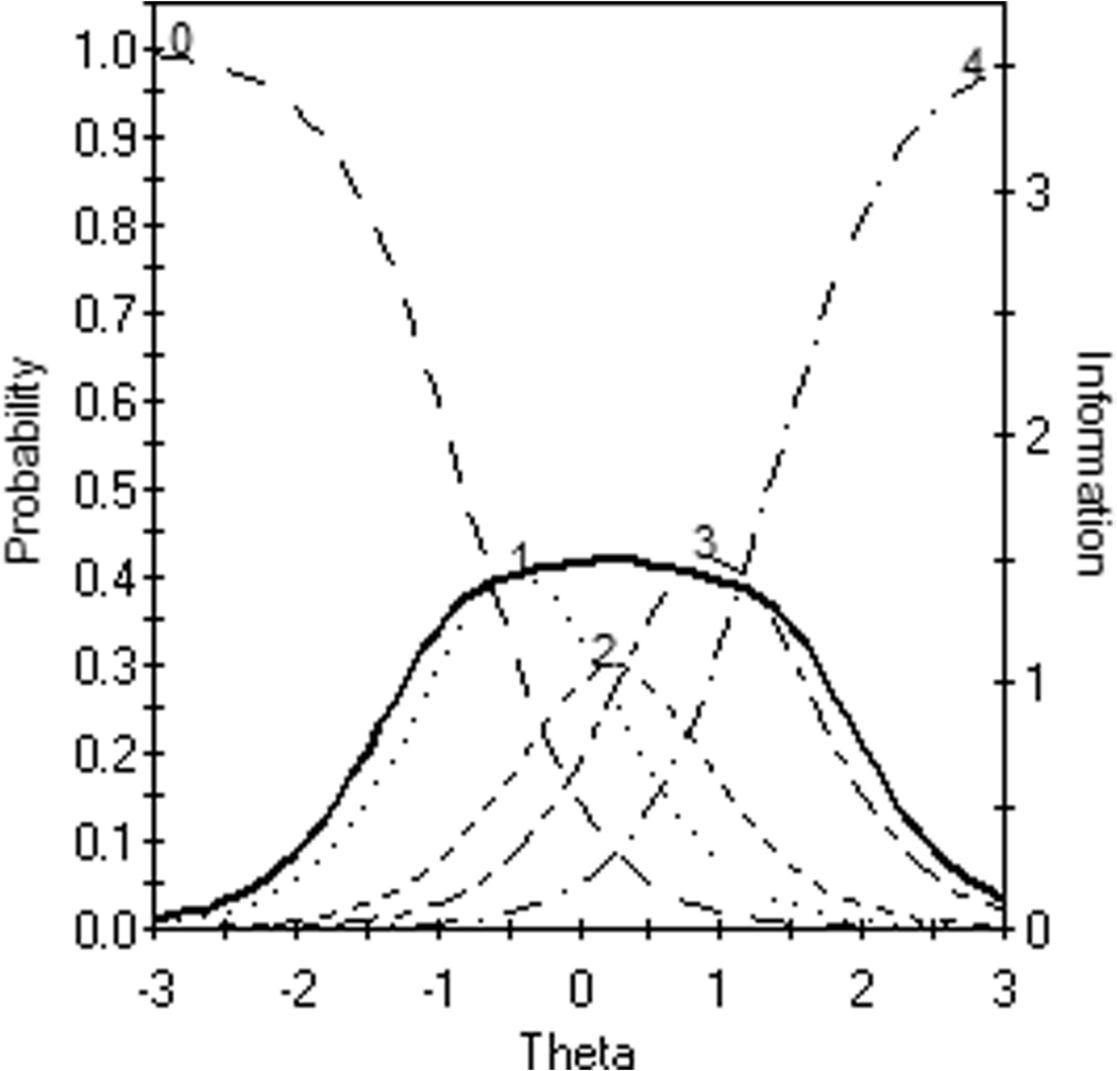
Category response curves (CRCs; 0–4) and item information curve (straight line) Item 17, *Feeling down or depressed* (GP clients). X-axis: Position on the latent distress continuum; left y-axis: Probability of endorsement; right y-axis: Information provided by item 17. The straight line depicts the information provided by this item conditional on theta (x-axis). The other lines depict the CRCs for this item, that is the probability of endorsing a specific response category, conditional on theta: 0: ‘Not present’, 1: ‘Sometimes’, 2: ‘Regularly’, 3: ‘Often’, & 4: ‘very often / constantly present’

To further illustrate this, Fig. 1 displays the five OCCs for item 17 (*Feeling down or depressed*). In this figure, the x-axis represents the amount of distress that is experienced by respondents. This metric of this dimension may be conceived as approximately standard-normal. The bold line represents the information that this item provides for differentiating respondents based on distress. Because information is additive under IRT models, these functions may be summed to form the so-called Test Information Functions (TIFs), from which local standard errors functions of person estimates can be deduced.

In order to compare various indicators of model fit of both scoring options, we first compared observed with expected item score frequencies using the *S-X*^2^ item-fit statistic proposed by Orlando and Thissen [33]. Second, we compared the mean value of the exact test probabilities for each scoring rule across items, and third, we compared the RMSEAs (with lower values indicating better model fit) of both scoring rules.

Furthermore, in order to get an impression of the usefulness of the five response options for each item, we investigated the spread of response categories by computing the smallest distances between threshold parameters within items. Additionally, we used the item parameters derived from the calibration sample to compute IRT-scores for the clients in the CV- and in the MISS samples. Finally, under the assumption of acceptable model fit for both scoring rules, we used the standard error functions to compare local measurement precision.

All IRT-analyses were performed using IRTPRO 3 [34].

## Results

### Model fit and measurement precision

The results of the tests that compare observed and expected item score frequencies can be found in Table 1 (0–2) and Table 2 (0–4). Note that the lower the probability of the test statistic, the worse model fit is.

**Table 1:** Item-wise chi-square tests of model fit (0–2)

Order	Item stem (abbreviated)	X^2^	df	Probability
17	Feeling down or depressed	53.63	46	0.2045
19	Worry	29.74	45	0.9613
20	Disturbed Sleep	53.44	51	0.3799
22	Listlessness	73.23	46	0.0065
25	Tense	39.48	43	0.6254
26	Easily irritated	32.59	48	0.9566
29	That you just can’t do anything anymore	46.59	40	0.2191
31	(…) take any interest in the people and things around you	41.73	38	0.3113
32	That you can’t cope anymore	31.59	38	0.7598
36	That you can’t face it anymore	52.79	34	0.0209
37	No longer feel like doing anything	64.36	38	0.0048
38	Have difficulty in thinking clearly	39.67	47	0.7677
41	Did you easily become emotional	46.94	48	0.5171
48	(…) to put aside thoughts about any upsetting event(s)	61.17	48	0.0958

**Table 2:** Item-wise chi-square tests of model fit (0–4)

Order	Item stem (abbreviated)	Χ^2^	df	Probability
17	Feeling down or depressed	161.66	144	0.1490
19	Worry	144.45	143	0.4509
20	Disturbed Sleep	203.09	169	0.0377
22	Listlessness	177.70	145	0.0335
25	Tense	133.39	136	0.5478
26	Easily irritated	134.17	150	0.8186
29	That you just can’t do anything anymore	132.26	121	0.2278
31	(…) take any interest in the people and things around you	155.47	121	0.0189
32	That you can’t cope anymore	123.85	115	0.2696
36	That you can’t face it anymore	173.99	113	0.0002
37	No longer feel like doing anything	153.52	116	0.0112
38	Have difficulty in thinking clearly	131.36	152	0.8857
41	Did you easily become emotional	191.04	156	0.02
48	(…) to put aside thoughts about any upsetting event(s)	189.36	173	0.1870

For the 0–2 scoring rule, item 22 (*Listlessness*) and item 37 (*No longer feel like doing anything)* had *p*-values that were smaller than .01, and item 36 (*Can’t face it anymore*) had *p* < .05. For the 0–4 scoring rule, item 36 had *p* < .01, and five other items had p < .05. However, with large sample sizes, the tests of model fit for individual items are very powerful tools to detect even slight deviations between observed and expected item scores [34].

To get an impression of overall model fit, we calculated the mean value of the exact test probabilities for each scoring rule across items (last column Tables 1 and 2). These indicated relatively poorer model fit for the scoring rule with five response options (.262) than for the scoring rule with only three response options (.416). However, the RMSEAs of both scoring rules were nearly identical: .04 for the scoring rule with five response options and .05 for the scoring rule with three response options. To conclude, in line with earlier research findings regarding the effect of the number of response options on model fit, we found poorer model fit for the 0–4 scoring rule compared to the 0–2 scoring rule. However, the data of both scoring options may be adequately modelled by graded response models.

**Fig. 2:**
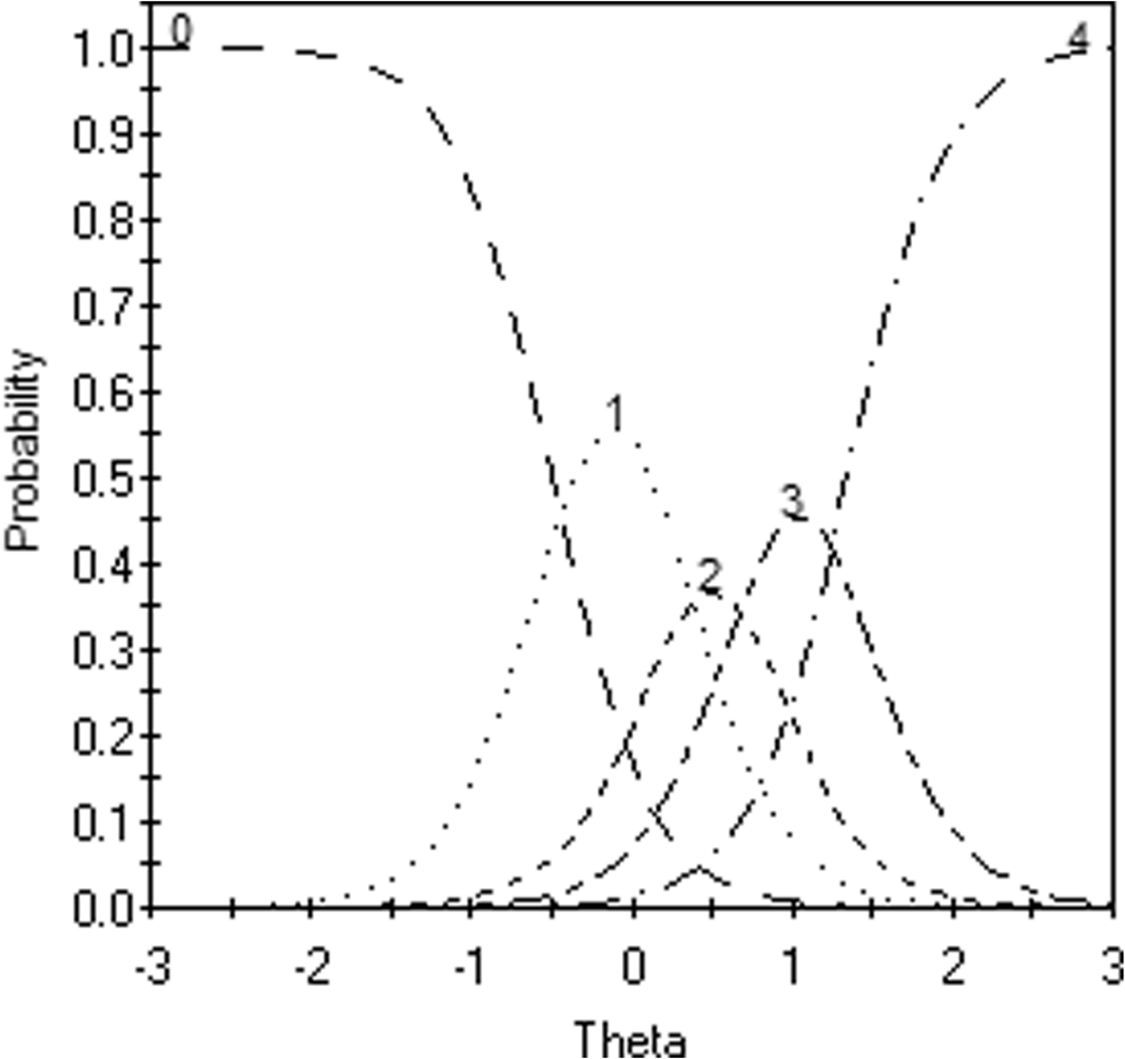
Category response curves (CRCs; 0–4) and item information curve (straight line) Item 32, *That you can’t cope anymore*. X-axis: Position on the latent distress continuum; left y-axis: Probability of endorsement; The lines depict the CRCs for this item, that is the probability of endorsing a specific response category, conditional on theta: 0: ‘Not present’, 1: ‘Sometimes’, 2: ‘Regularly’, 3: ‘Often’, & 4: ‘Very often / constantly present’

Inspection of the OCCs for all items showed that the distance between the mid-thresholds (b_12_ and b_23_) was always smaller than the distance between the first and second threshold (b_01_ and b_12_), or between the third and fourth threshold (b_23_ and b_34_). This indicated that the response option ‘Regularly’ in between ‘Sometimes’ and ‘Often’ has little practical value, and that differentiating between the two highest response categories ‘Often’ and ‘Constantly present’ seems advisable. To illustrate this, Fig. 2 shows the OCCs of item 32, *That you can’t cope anymore*.

For this item, the third response option (denoted by 2) is practically redundant, because nearly all the surface under its curve is shared with the second (1) and the fourth (3) response option. For nearly all levels of distress (except those that are very close to θ = .47), other response options are always more likely than the third response option.

**Fig. 3:**
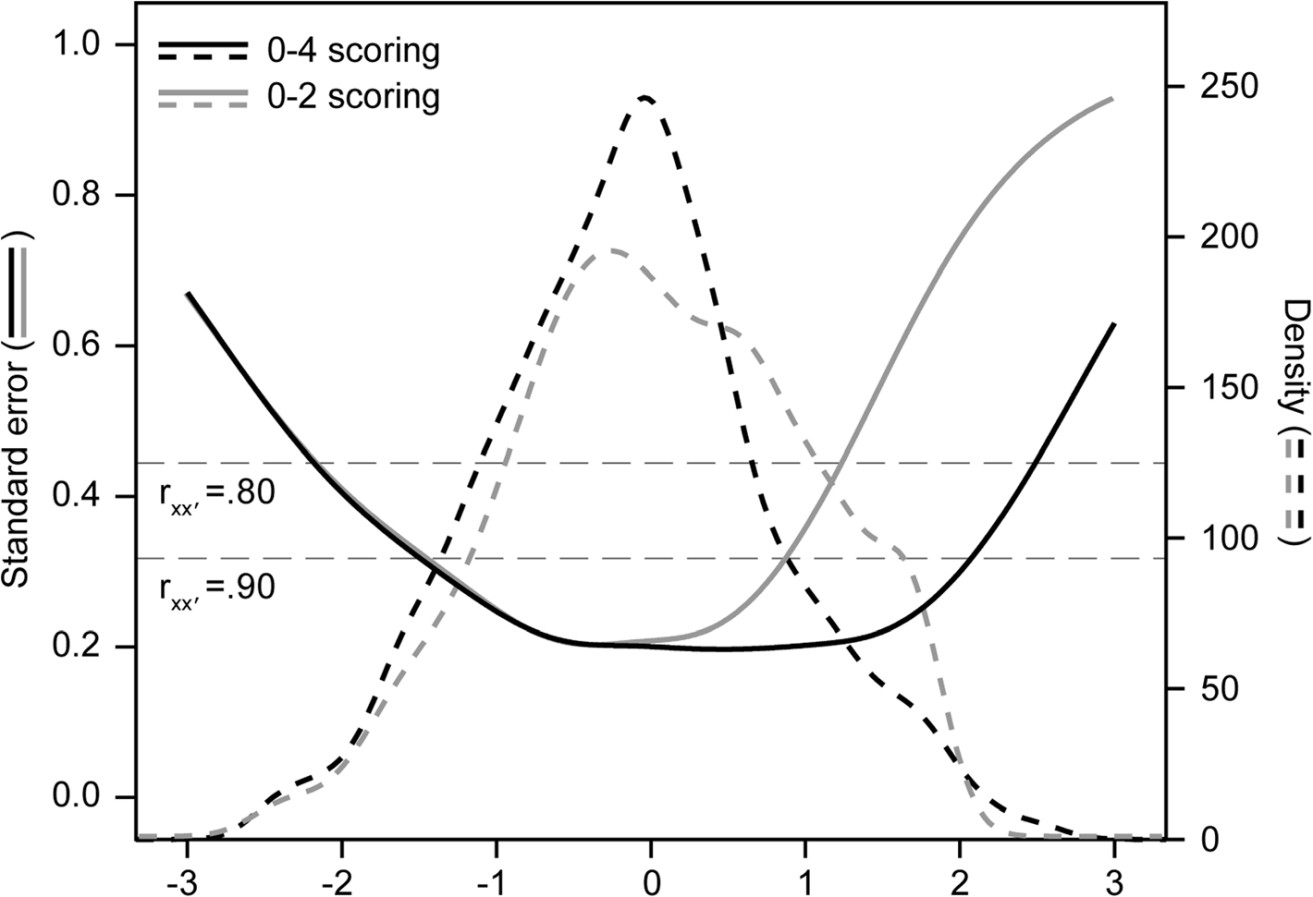
Standard error functions and population densities for both scoring options

The standard error functions of both scoring rules are displayed in Fig. 3. These are nearly identical in the range of theta = − 3 to 0. For higher levels of distress though, the standard errors for the scoring rule with three response options (green line) are approximately 50% larger than the standard errors for the scoring rule with five response options. So, for medium and high levels of distress, the 0–4 scoring results in higher measurement precision than the 0–2 scoring.

### Convergent and discriminant validity

As shown in Table 3, both scoring rules yield approximately the same correlation coefficients with other nonspecific indicators of mental health. In addition, both scoring rules are equally strongly related to the construct of Neuroticism. Thus, the indicators of convergent validity were slightly in favor of the 0–4 scoring rule, and with respect to discriminant validity, both scoring options performed equally well.

**Table 3:** Convergent and discriminant validity of the three- and five-point Likert scales

	MQ	GHQ	Neuroticism
Distress 0–2	−.642**	.536**	.543**
Distress 0–4	−.662**	.555**	.550**

MQ: Maastricht Vital Exhaustion Questionnaire, GHQ: General Health Questionnaire, Distress 0–2: Recoded item scores with three response options, Distress 0–4: Original item scores with five response options; ** p > .01, *p > .05

### Predictive validity

To compare the predictive power of both scoring rules for obtrusion of daily chores and activities (‘No problems’ versus ‘Some problems’/’ Unable to perform’), we conducted logistic regressions with the distress scores of both scoring options at baseline as predictors. As Table 4 shows, for short-term prediction, the five-point rating scale was slightly superior, but both scoring rules performed approximately equally well for long-term prediction.

**Table 4:** Results logistic regressions for prediction of obtrusion of daily chores and activities

		Χ^2^	df	p	Nagelkerke R^2^
Short term	**DIS**_**0–2**_	10.9	1	.001	.050
	**DIS**_**0–4**_	15.0	1	.001	.071
Long term	**DIS**_**0–2**_	4.5	1	.035	.023
	**DIS**_**0–4**_	3.9	1	.047	.021

Distress 0–2: Recoded item scores with three response options, Distress 0–4: Original item scores with five response options

From Table 5, it can be deduced that both scoring rules performed very similar in terms of predicting relevant futures outcomes. In case there was a difference, the 0–4 scoring of item scores generally performed better than the 0–2 scoring. The differences in the size of Pearson correlations were equal or less than .03 though. Interestingly, in predicting days of sick leave (computed as days from sick notice till start of reintegration), only the five-point scoring rule resulted in a significant finding, where the three-point scoring rule did not. Thus, the differences between the two scoring rules in predicting relevant future outcome measures were generally quite small, although in all cases, the 0–4 scoring rule was slightly superior to the 0–2 scoring rule.

**Table 5:** Predictive validities (Pearson correlations) distress scale for various outcome measures of social and occupational functioning

		BIOPRO-R^3^	BIOPRO-G^4^	UBOS-EXH^5^	UBOS-DIS^6^	UBOS-COM^7^	Sick- leave
Shortterm^1^	**DIS**_**0-2**_	.253**	.305**	–	–	–	–
	**DIS**_**0–4**_	.253**	.328**	–	–	–	–
Longterm^2^	**DIS**_**0–2**_	.260**	.317**	.145**	.074	.117*	.122
	**DIS**_**0–4**_	.259**	.321**	.173**	.088	.138*	.140*

^1^: Six month, ^2^: Twelve month, ^3^: Selected relational problem statements Biographical problem list, ^4^: Selected general problem statements Biographical problem list, ^5–7^: UBOS scales Exhaustion, Distance and Competence, ** *p* > .01, *p > .05, distress 0–2: Recoded item scores with three response options, distress 0–4: Original item scores with five response options

## Discussion

### Main findings

Although collapsing the three highest response alternatives did improve model fit, model fit of items with five response alternatives was still acceptable. Inspection of the spread of response alternatives indicated that in case of the 4DSQ, it is rather the mid category (*Regularly*) that seems to be redundant, and not one or two of the highest response options. Furthermore, with respect to local measurement precision, the five-point Likert scale was clearly advantageous for medium and high levels of distress. However, the gain in measurement precision did not result in substantial gains in various indices of scale validity. The differences in correlation coefficients that we found were less than .03. Still, for effects that are near the threshold of significance, as prediction of days of sick leave in our study, using the original five-point Likert response scale may reveal effects that the three-point Likert response scale does not reveal. In addition, using the three-point Likert response scale did not lead to a higher discriminant validity of the scale. That is, the correlation of both types of distress scores with Neuroticism were nearly equal.

### Strengths and limitations

To our knowledge, this was the first study that investigated the effect of the type of response scale on multiple indicators of various types of validity. In addition, for some indicators of predictive validity, we could compare short-term (six month) and long-term (twelve months) predictions of both scoring rules.

The main limitation of this study was that the data of the three-point Likert scale were not obtained using three response alternatives. Thus, we cannot state that using the original five response alternatives is the best way to collect data for the distress items of the 4DSQ in general.

Another minor limitation was that we had to remove two out of sixteen items because these violated one of the IRT assumptions. However, because the items that had to be removed correlated highly with the other item of the pair (.80–.90), we may argue that little item-specific information is lost by removing these two items.

Further note that our claim, that for all criterion measures except Neuroticism, higher correlation coefficients would be indicative of the more valid scoring rule is somehow disputable for the UBOS dimensions. This is because the UBOS is scored on a seven-point Likert scale, which might also trigger the response set of extreme responding in respondents. Thus, an alternative explanation for the fact that the 0–4 scoring rule correlates higher with the UBOS dimensions than the 0–2 scoring rule could be that both sets of scores (DIS0–4 and UBOS) are contaminated by the response style of extreme responding, causing an inflated correlation coefficient.

Furthermore, two of the samples used in this study contained adolescents, the calibration sample (N_adolescents_ = 46) and the convergent validity sample (N_adolescents_ = 1). An article that reports findings on the measurement invariance of the 4DSQ scales with respect to age (adolescents, 10–17; ‘emerging adults’, 18–25; and adults, 26–40) is currently in preparation. We may report that the 4DSQ scale scores are not biased by age. In addition, it should be noted that the convergent validity sample contained 55 respondents only, and that such a sample size only warrants tentative conclusions.

We also want to acknowledge that our choice for the Neuroticism scale of the NEO-FFI as an indicator of discriminant validity is disputable from a theoretical point of view [35].

(Ploubidis & Frangou, 2011). cf both scoring rules with Neuroticism were approximately as high as the correlation coefficients of both scoring rules with the second indicator of convergent validity (the GHQ).

## Conclusion

In conclusion, for cross-sectional research, it does not seem to matter very much whether the item scores are recoded or not. In any case, this study suggests that using the original five category response data is never disadvantageous. For both clinical applications and longitudinal research applications where the interest is in monitoring scores of individuals over time, the response scale with five categories is to be preferred. This is because in these settings, the increased measurement precision of the five-point Likert scale for medium and high levels of distress will probably lead to a better measurement of change. For example, between baseline- and post-treatment measures of distress. Thus, our recommendation is that scoring should be based on the original response scales with five response options.

### Directions for future research

In order to get an impression of whether our findings may de generalized to domains other than distress, the analyses conducted for this article could be replicated with data gathered with the items of the other three 4DSQ domains: anxiety, depression, and somatoform symptoms.

## Appendix

**Table 6:** Chosen BIOPRO problem statements

Facet	Content Statement
Relational	Parents
Relational	Partner
Relational	(Own) children
Relational	Other relevant persons
Relational	Other persons in general
Relational	Loneliness
General	Financial
General	Housing
General	Study
General	Work
General	Self-concept
General	Living conditions
General	Worrying
General	Other problem not mentioned in statements

**Table 7:** Descriptive Statistics distress scale at baseline, after six months (short-term), and after twelve months (long-term)

Moment	Scale	Min	Mean	SD	Max
Baseline	Distress	1	2.51	0.87	5
Short-term	Distress	1	1.92	0.84	4.93
Short-term	BIOPRO-R	7	10.75	1.4	12
Short-term	BIOPRO-G	9	13.78	1.71	16
Long-term	Distress	1	1.78	0.78	4.86
Long-term	BIOPRO-R	6	10.61	1.43	12
Long-term	BIOPRO-G	8	13.95	1.75	16
Long-term	UBOS-EXH	0	2.52	1.66	6
Long-term	UBOS-DIS	0	1.9	1.53	6
Long-term	UBOS-COM	0	3.86	1.25	6

## Abbreviations

MISS study: Minimal Intervention for Stress-related mental disorders with Sick leave
4DSQ: Four-Dimensional Symptom Questionnaire
BPL: Biographical Problem List
CRC: Category Response Curve
CV: Convergent validity
GHQ: General Health Questionnaire
GP: General Practitioner
GRM: Graded Response Model
IRT: Item Response Theory
MQ: Maastricht Vital Exhaustion Questionnaire
NEO-FFI: NEO Five Factor Inventory
OCC: Operating Characteristic Curve
RMSEA: Root Mean Squared Error of Approximation
SE: Standard Error
TCC: Test Characteristic Curve
TIF: Test Information Functions
UBOS: Utrecht Burnout Scale
